# Mitochondrial thiol oxidase Erv1: both shuttle cysteine residues are required for its function with distinct roles

**DOI:** 10.1042/BJ20131540

**Published:** 2014-05-13

**Authors:** Swee Kim Ang, Mengqi Zhang, Tiziana Lodi, Hui Lu

**Affiliations:** *Manchester Institute of Biotechnology, Faculty of Life Sciences, University of Manchester, 131 Princess Street, Manchester M1 7DN, U.K.; †Department of Life Sciences, University of Parma, I-43100 Parma, Italy

**Keywords:** CXXC motif, mitochondrial import, mitochondrial import and assembly pathway (MIA pathway), thiol oxidase, ALR, augmenter of liver regeneration, AMS, 4-acetamido-4′-maleimidylstilbene-2,2′-disulfonic acid, *At*Erv1, *Arabidopsis thaliana* Erv1, Cox17, cytochrome *c* oxidase 17, CTC, charge-transfer complex, Erv, essential for respiration and viability, hMia40, human Mia40, IAM, iodoacetamide, IMS, intermembrane space, *Lt*Erv, *Leishmania tarentolae* Erv, MIA, mitochondrial import and assembly, mBBr, monobromobimane, mmPEG24, methyl-PEG24-maleimide, NEM, *N*-ethylmaleimide, *Pf*Erv, *Plasmodium falciparum* Erv, TCA, trichloroacetic acid, TCEP, tris-(2-carboxyethyl)phosphine, Tim, translocase of the inner membrane, TPI1, triose phosphate isomerase 1, TRP1, tryptophan requiring 1, WT, wild-type

## Abstract

Erv1 (essential for respiration and viability 1), is an essential component of the MIA (mitochondrial import and assembly) pathway, playing an important role in the oxidative folding of mitochondrial intermembrane space proteins. In the MIA pathway, Mia40, a thiol oxidoreductase with a CPC motif at its active site, oxidizes newly imported substrate proteins. Erv1 a FAD-dependent thiol oxidase, in turn reoxidizes Mia40 via its N-terminal Cys^30^–Cys^33^ shuttle disulfide. However, it is unclear how the two shuttle cysteine residues of Erv1 relay electrons from the Mia40 CPC motif to the Erv1 active-site Cys^130^–Cys^133^ disulfide. In the present study, using yeast genetic approaches we showed that both shuttle cysteine residues of Erv1 are required for cell growth. In organelle and *in vitro* studies confirmed that both shuttle cysteine residues were indeed required for import of MIA pathway substrates and Erv1 enzyme function to oxidize Mia40. Furthermore, our results revealed that the two shuttle cysteine residues of Erv1 are functionally distinct. Although Cys^33^ is essential for forming the intermediate disulfide Cys^33^–Cys^130^′ and transferring electrons to the redox active-site directly, Cys^30^ plays two important roles: (i) dominantly interacts and receives electrons from the Mia40 CPC motif; and (ii) resolves the Erv1 Cys^33^–Cys^130^ intermediate disulfide. Taken together, we conclude that both shuttle cysteine residues are required for Erv1 function, and play complementary, but distinct, roles to ensure rapid turnover of active Erv1.

## Abstract 2

Erv1 is a sulfydryl oxidase, an essential component of mitochondrial MIA pathway. The present study shows that both shuttle cysteine residues of Erv1 are required for its function, they play complementary, but distinct, roles to ensure rapid turnover of active enzyme.

## INTRODUCTION

Disulfide bond formation is important for folding and function of many proteins, and it plays an essential role during the biogenesis of mitochondrial IMS (intermembrane space) proteins [1–4]. In *Saccharomyces cerevisiae*, all the mitochondrial IMS proteins are encoded by the nuclear genome. After protein translation in the cytosol, many of the IMS precursor proteins are imported into mitochondria through the IMS MIA (mitochondrial import and assembly) pathway, which consists of two essential components, Mia40 and a FAD-dependent thiol oxidase Erv1 (essential for respiration and viability 1) [1–3]. Most substrates of the MIA system are small cysteine-rich proteins with conserved twin CX_3_C [e.g. Tim9 (translocase of the inner membrane) and Tim10] or twin CX_9_C [e.g. Cox17 (cytochrome *c* oxidase 17) and hMia40 (human Mia40)] motifs. These substrates are imported into the mitochondrial IMS in a cysteine-reduced unfolded conformation [5–7]. Upon import into the mitochondria, they interact with oxidized Mia40 and form an intermolecular disulfide-bonded substrate–Mia40 complex, leading to disulfide transfer from Mia40 to the substrate and thus trapping the oxidized substrate in the IMS.

All Mia40 proteins contain three pairs of disulfide bonds in a highly conserved C-terminal domain. Although the two disulfides formed between the twin CX_9_C motifs are stable and play a structural role, a redox-active disulfide bond is formed between cysteine residues in the CPC motif, e.g. Cys^296^–Cys^298^ in yeast Mia40 [8–10]. The structures of yeast and hMia40 revealed the presence of a hydrophobic binding cleft in close proximity to the CPC motif redox active site, which plays an important role in driving protein–protein interaction and the recognition of specific substrates [9,10]. Structural data suggest that the second cysteine residue of the CPC motif is more exposed than the first cysteine on the hydrophobic binding cleft, and is therefore better positioned for covalent interaction with its substrate proteins. Consistently, several mixed disulfide intermediates have been identified, forming between the second cysteine of the CPC motif and a docking cysteine of substrate proteins (e.g. the small Tim proteins and Cox17) [10,11]. Upon disulfide transfer to substrate protein, the Mia40 CPC motif is reduced. Subsequently, the reduced Mia40 is reoxidized by Erv1 to restore its function. Thus Mia40 is a substrate (electron donor) of Erv1; Erv1 assists and catalyses the Mia40-dependent import and oxidative protein folding in the mitochondrial IMS. It is worth mentioning that, although a fraction of the Mia40 CPC motif binds iron or an iron–sulfur cluster *in vivo*, this cofactor-containing Mia40 seems not to act as electron donor for Erv1 [12].

**Figure 1:**
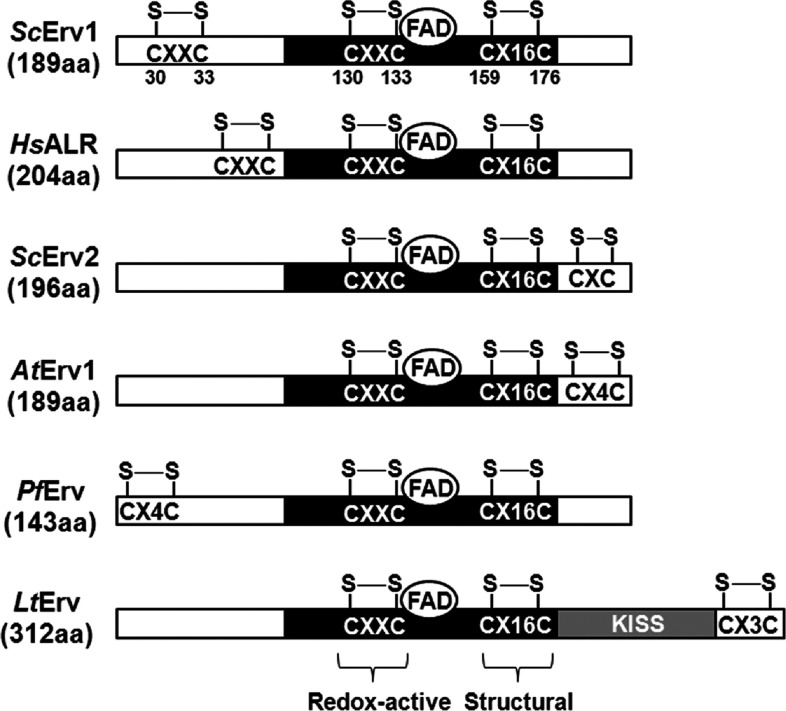
Schematic sequence alignment of ERV/ALR homologues The conserved FAD-binding catalytic domain (black) contains a redox-active CXXC disulfide and a structural CX_16_C disulfide. An additional shuttle disulfide is located at non-conserved domain (white). The architecture of *Lt*Erv is slightly altered with a KISS (Kinetoplastida-specific second) domain (grey) of approximately ~200 amino acids at the C-terminus. *Hs*, *Homo sapiens*; *Sc*, *S. cerevisiae.*

Erv1 belongs to the single domain ERV/ALR thiol oxidase subfamily, including the mammalian homologue ALR (augmenter of liver regeneration), plant Erv1 [*At*Erv1 (*Arabidopsis thaliana* Erv1)], yeast Erv2 [*Sc*Erv2 (*S. cerevisiae* Erv2)] of the ER (endoplasmic reticulum) lumen and other Erv proteins (Figure 1). ERV/ALR enzymes are characterized by a highly conserved FAD-binding catalytic core domain of approximately 100 amino acids containing a CXXC redox active-site disulfide (Cys^130^–Cys^133^ in Erv1) located proximally to the isoalloxazing ring of the FAD cofactor (also known as proximal disulfide) and a C-terminal CX_16_C structural disulfide (Cys^159^–Cys^176^ in Erv1). In addition, there is a third disulfide bond (Cys^30^–Cys^33^ in Erv1) located either on the N- or C-terminus of a non-conserved range (distal disulfide), which functions to transfer electrons from reduced Mia40 or equivalents to the redox active-site disulfide, thus also referred to as shuttle disulfide [13–16]. The cysteine motif of the distal (shuttle) disulfide is highly variable phylogenetically, varying from CRXC at the N-terminus of Erv1 and ALR, CX_4_C at the N-terminus of *Pf*Erv (*Plasmodium falciparum* Erv), to CXC, CX_3_C and CX_4_C at the C-terminus of Erv2, *Lt*Erv (*Leishmania tarentolae* Erv) and *At*Erv1 respectively (Figure 1). Also, the enzyme architecture of Kinetoplastida ERV homologues (e.g. *Lt*Erv) is slightly altered with an additional C-terminal domain of ~200 amino acids with unknown function [16]. Several structures from the core domain of ERV/ALR proteins are available, all of which crystallized as head-to-tail homodimers [17–20]. Each subunit contains a four-helical bundle that harbours the FAD cofactor and an additional (fifth) single-turn helix.

The interaction between Mia40 and Erv1/ALR remained unclear until a study revealed that ALR shares the same Mia40 hydrophobic binding cleft with Mia40 substrate proteins [21]. Consistently, a recent study based on a full-length Erv1C30S/C133S mutant showed the presence of an amphipathic helix close to the shuttle cysteine residue and that it was important for the recognition of Mia40 [17]. The current model for the Erv1 enzyme mechanism is that Erv1 oxidizes the reduced Mia40 CPC motif through the N-terminal Cys^30^–Cys^33^ shuttle disulfide, which is then reoxidized by transferring two electrons to the proximal active-site Cys^130^–Cys^133^ disulfide, and then the electrons are relayed to cytochrome *c* or molecular oxygen through the bound FAD for regeneration of Erv1 activity [13,22–26].

However, it was unclear how exactly the two shuttle cysteine residues (Cys^30^ or Cys^33^) of Erv1 relay electrons from the Mia40 CPC motif to the active-site disulfide. Which cysteine residue of Erv1 forms the intermolecular disulfide bond with Mia40 and whether they play overlapping or different roles in the enzymatic function are questions still remaining to be addressed. For human ALR, a gel-based analysis showed that both shuttle cysteine residues interacted equally well with the second cysteine residue of the hMia40 CPC motif [27], indicating that both shuttle cysteine residues have an overlapping function. On the other hand, based on the structure, the 2nd shuttle cysteine residue (analogous to Erv1 Cys^33^) of ALR was proposed to interact preferentially with hMia40 since it is closely followed by a stretch of hydrophobic residues responsible for substrate binding [27]. In yeast, in the case of Erv2, it was shown that both shuttle cysteine residues were required for its function *in vivo*, and both single cysteine mutants can form a disulfide bond with the active-site cysteine [14]. In the case of Erv1, although Cys^33^ is essential for cell viability, Cys^30^ seems dispensable under certain conditions [23,28]. Thus Cys^33^ of Erv1 was proposed to form an intermolecular disulfide bond with Mia40.

In the present study, using a combination of *in vivo*, in organelle and *in vitro* methods, we showed that both shuttle cysteine residues of Erv1 are essential for its function. They are both required for yeast cell growth, mitochondrial import of MIA substrates and Erv1 oxidase activity in the *in vitro*-reconstituted Mia40–Erv1 systems. Furthermore, our results showed that the two shuttle cysteine residues of Erv1 are functionally distinct; whereas Cys^30^ interacts and receives electrons directly from the Mia40 CPC motif, Cys^33^ transfers electrons gained through Cys^30^ to the active-site disulfide via formation of a Cys^33^–Cys^130^ intermediate disulfide bond. We also showed that Cys^30^ and Cys^33^ of Erv1 have the same p*K*_a_ values and thus similar nucleophilicity despite their distinct roles in the enzymatic mechanism. Our results are consistent with the view that steric restrictions play a crucial role in thiol–disulfide exchange reactions, which mediate the kinetics and substrate specificity of the thiol oxidase enzymes.

## EXPERIMENTAL

### Cloning and mutagenesis

A construct encoding C-terminally LE(H)_6_-tagged full-length WT (wild-type) Erv1 cloned into *Escherichia coli* expression vector pET24a(+) (Novagen) using the Nde1 and Xho1 restriction sites was used as DNA template [29] to generate all mutant constructs for protein purification work in the present study. For yeast studies, *ERV1* together with endogenous promoter and terminator regions were amplified by PCR from the genomic DNA of the WT CY4 strain [30], followed by insertion into centromeric plasmids pRS413 and pRS414 via the Xho1 and BamH1 restriction sites. Cysteine-to-serine mutations were introduced by PCR site-directed mutagenesis (Stratagene) using overlapping primer pairs containing the desired mutation point(s). Correct sequences of all WT and mutant constructs were verified by DNA sequencing.

### Complementation assays

A *S. cerevisiae* strain harbouring the *ERV1* gene under the *GAL10* promoter [31] was transformed with *TRP1* (tryptophan requiring 1)-containing plasmids pRS414 with or without WT Erv1 or cysteine mutants. The strains were grown on a synthetic selective medium containing glycerol (3%) supplemented with glucose (0.2%) or galactose (0.2%) in order to repress or induce the chromosomal *ERV1* allele. The strains were first grown in the presence of galactose, and then shifted to a medium containing glucose for 48 h before spotting on to agar medium contai-ning glucose. For plasmid shuffling tests, a ∆*erv1*-knockout strain containing a copy of WT *ERV1* on the *URA3* plasmid [32] was co-transformed with the *HIS3* plasmid pRS413 with or without WT or mutant *ERV1* and *TRP1* plasmid pRS414 with mutant *ERV1*. Following selection, the strains were analysed on 5-fluoro-orotic acid plates to counterselect against WT *ERV1* on URA3-containing plasmid. Plates were incubated for up to 3 days at 25°C, 30°C or 37°C.

### Protein purification

WT Erv1 and cysteine residue mutants were expressed in the *E. coli* strain Rosetta-gami™ 2 (Novagen). The procedure for LE(H)_6_-tagged protein purification was as described previously [13]. Briefly, protein expression was induced in the presence of 0.5 mM IPTG and 10 μM FAD at 16°C for 16–20 h. The induced cell pellets were resuspended in buffer A [150 mM NaCl and 50 mM Tris/HCl (pH 7.4)] supplemented with 5 mM imidazole, 50 μM FAD and one tablet of EDTA-free protease inhibitor cocktail (Roche) and then sonicated on ice. The supernatant fraction was loaded on a column containing 2–3 ml of Ni^2+^-charged His. Bind resin (Novagen) pre-equilibrated with binding buffer. The column was washed with 20–30 ml of wash buffer (buffer A containing 20 mM imidazole) before the protein was eluted with 4–6 ml of elution buffer (buffer A containing 500 mM imidazole). Eluted proteins were supplemented with 100 μM FAD and stored in −80°C until further use. For GST-tagged Mia40c (amino acids 284–403), the purification procedure was as described previously [8]. Briefly, protein was bound to 2–3 ml of glutathione–Sepharose 4B beads (GE Healthcare) overnight with gentle rotation at 4°C in buffer A. Unbound materials were removed by washing the beads with 20–30 ml of ice-cold buffer A. Protein elution was achieved by overnight incubation at 4°C in 4–6 ml of cleavage buffer (20–25 units/ml thrombin in buffer A) to remove the GST tag. All affinity-purified proteins were further purified by size-exclusion chromatography using a Superdex 75 10/30 column connected to an ÄKTA FPLC system (GE Healthcare) at 4°C in buffer A.

### Mitochondria isolation

The *P_GAL_-ERV1* strains were grown in liquid selective medium containing glycerol (3%) and glucose (0.2%) for 48–72 h with aeration at 30°C. Mitochondria isolation was performed as described previously [33].

### Protein steady-state level and submitochondrial localization analyses

Total mitochondrial proteins were separated on Tris/Tricine SDS/PAGE [34] before Western blotting and immunodecoration with antibodies against the respective protein of interest. For protein submitochondrial localization, mitochondria (50 μg) were treated with proteinase K (0.2 mg/ml) in the presence of SM buffer [0.6 M sorbitol and 20 mM MOPS (pH 7.4)], hypotonic buffer [20 mM MOPS (pH 7.4)] or lysis buffer [1% (w/v) digitonin and 20 mM MOPS (pH 7.4)] as described in [35]. Proteolysis was inhibited by 4 mM PMSF. Samples were TCA (trichloroacetic acid)-precipitated before analysis by Tris/Tricine SDS/PAGE (16% gel) and Western blotting.

### Thiol-trapping assays

*In vitro* redox time course reactions were quenched using IAM (iodoacetamide; 20 mM) or AMS (4-acetamido-4′-maleimidylstilbene-2,2′-disulfonic acid; 5 mM). For mitochondrial samples, protein thiols were alkylated with mmPEG24 (methyl-PEG24-maleimide; 2 mM). The thiol alkylation agents were prepared in 2× non-reducing SDS/PAGE sample buffer and mixed with an equal volume of protein samples at the designated time points or added to TCA-precipitated protein pellets. Samples were incubated in the dark for 30–60 min at room temperature (20°C) before resolving by Tris/Tricine SDS/PAGE (16% gel). For redox state analysis *in vivo*, cells were grown in glucose or galactose medium until the mid-exponential phase before incubation with 25 mM NEM (*N*-ethylmaleimide) to alkylate protein thiols *in situ* on ice for 30 min. Total cellular protein was extracted then using non-reducing SDS/PAGE sample buffer and glass beads, and separated by SDS/PAGE (16% gel) followed by Western blot analysis.

### Oxygen consumption assays

Erv1 oxidase activity was measured using a Clark-type oxygen electrode (Hansatech Instrument) in a 0.5 ml reaction volume at 25°C in buffer A containing 1 mM EDTA as described previously [13]. Data analysis of the oxygen consumption profile and the calculation of reaction slope were performed using the Microcal™ Origin™ statistical software package.

### Determination of protein thiol p*K*_a_ values

The p*K*_a_ values of protein thiols were measured by reaction with mBBr (monobromobimane) and fluorescence detection as described in [36]. Buffers of constant ionic strength (*I*=0.15 M) independent of the pH at which they are used, as proposed in [37], were chosen. Two buffer systems with the pH range of 3.5–9.0 (15 mM acetic acid, 15 mM Mes and 30 mM Tris) and 5.7–10 (30 mM Aces, 15.6 mM Tris and 15.6 mM ethanolamine) were used. Both buffer systems also contained 120 mM NaCl and 0.1 mM DTPA (diethylenetriaminepenta-acetic acid). Purified proteins were buffer-exchanged to their respective buffer systems using NAP-5 columns (GE Healthcare) and adjusted to the desired pH values by using variable amounts of HCl or NaOH. The reactions were started by adding mBBr (2 μM) to protein solutions (2 μM) in total reaction volumes of 125 μl. The fluorescence intensity value change was monitored at λ_ex_=396 nm and λ_em_=482 nm for 30 min at 25°C using a Cary Eclipse fluorescence spectrophotometer. The initial rate of reaction was calculated using the points of at least the first 10 min of the reaction.

### Miscellaneous

Absorption spectra were measured using a Cary 300 spectrophotometer at 1-nm intervals from 250 to 700 nm and 1 cm quartz cells at 25°C as described in [13]. For multi-angle laser light scattering, protein samples were applied to a Superdex 200 10/30 gel-filtration column (GE Healthcare) running with buffer A. Proteins eluting from the column passed through an in-line DAWN EOS laser photometer set at 682 nm and an Optilab rEX refractor. To calculate the weight-averaged molecular mass, the light scattering intensity and eluent refractive index were analysed using ASTRA version 4.8 software.

## RESULTS AND DISCUSSION

### Both the shuttle cysteine residues of Erv1 are required for cell viability

**Figure 2:**
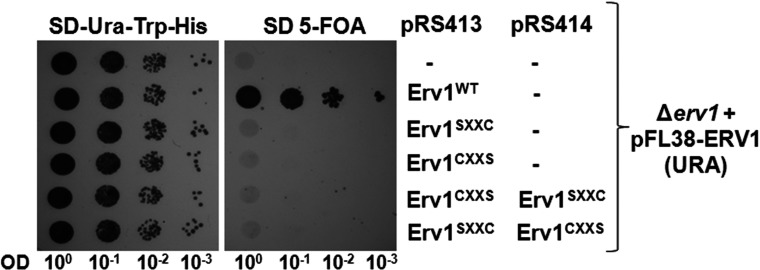
Both the N-terminal cysteine residues of Erv1 are required for cell viability Yeast complementation assays with Erv1^SXXC^ and Erv1^CXXS^ individually or co-expression via plasmid shuffling in the presence of 5-fluoro-orotic acid (FOA). Strains containing empty vector or WT Erv1 were used as negative and positive controls respectively. Cells were grown at 30°C for 3 days. OD, attenuance.

To investigate the requirement of individual shuttle cysteine residues of Erv1 in the protein function *in vivo*, we checked the cell viability by expressing plasmid-encoded Erv1 single cysteine-to-serine (C30S and C33S) mutants, hereafter named Erv1^SXXC^ and Erv1^CXXS^, in the *ERV1*-knockout strain ∆*erv1* that is isogenic to the WT W303 [32] (see the Experimental section; Figure 2). Upon counter-selection of WT *ERV1* on the *URA3*-containing plasmid, both mutants were unable to support cell growth at 30°C, suggesting that both Cys^30^ and Cys^33^ are required for yeast cell growth and thus the function of Erv1. Furthermore, co-expression of Erv1^SXXC^ and Erv1^CXXS^ also failed to rescue cell viability (Figure 2), indicating that these cysteine residues do not interact *in trans*, as they are required to be in the same CXXC motif in order to function. Our observation was different to the result previously published [23] where it was observed that only Erv1^CXXS^ was lethal and there was a WT-like cell growth at 30°C for Erv1^SXXC^ using the pYX142 overexpressing plasmid and a ∆*erv1*-knockout strain derived from the same genetic background. We did not observe any cell growth for Erv1^SXXC^ when the experiment was carried out at 25°C either (results not shown). It is probable that the discrepancy observed in the Erv1^SXXC^ complementation experiments between the present study and the one described previously [23] is due to the plasmid used and expression level of the protein. In the present study, the *ERV1* mutant was cloned in the centromeric plasmid pRS413, together with the endogenous promoter and terminator region. In [23], the mutant was cloned in pYX142 that is also a centromeric vector, but under the control of the promoter of the *TPI1* (triose phosphate isomerase 1) gene, encoding the abundant glycolitic enzyme Tpi1. Based on the global analysis of protein expression in yeast [38], Tpi1 protein level was estimated to be more than 2.07×10^5^ molecules/cell, whereas the Erv1 level was not detectable, meaning below the detection limit of 50 molecules/cell. Thus, under the control of the strong *TPI1* promoter, *Erv1^SXXC^* may act as a multicopy suppressor of the *Δerv1* growth-deficient phenotype, and can rescue the cell viability when it is overexpressed at a non-physiologically high level. In summary, our results suggest that both N-terminal shuttle cysteine residues of Erv1 are required for cell viability and. hence. Erv1 function providing that the mutant proteins were correctly imported into the mitochondria.

### The shuttle cysteine mutants of Erv1 are localized in the mitochondrial IMS

**Figure 3:**
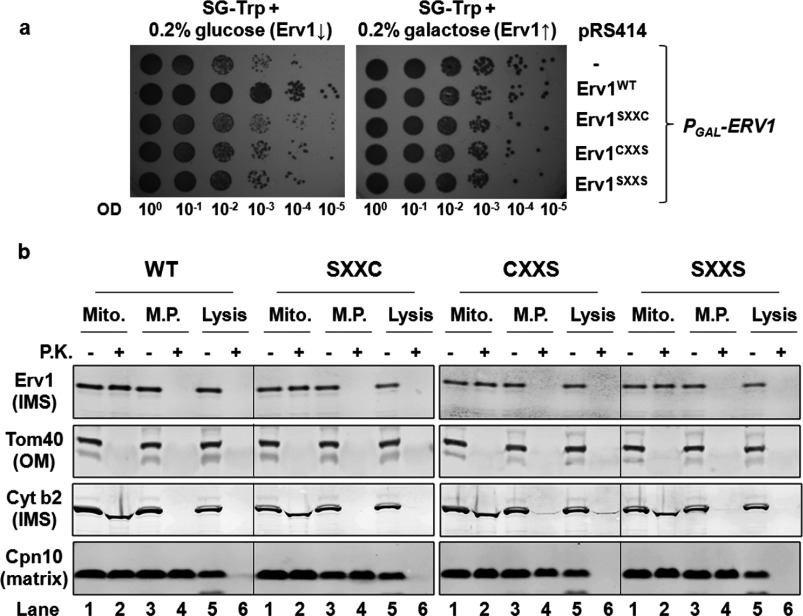
Mutation of the N-terminal cysteine residues had no effect on Erv1 localization in the mitochondrial IMS (**a**) Phenotype testing of yeast strains in which the endogenous *ERV1* is under the control of a *GAL10* inducible promoter were transformed with plasmid-encoded copies of the WT or shuttle cysteine mutants on glucose- or galactose-containing medium to repress (↓) or induce (↑) the expression of endogenous Erv1. The strain containing the empty vector served as a negative control. Cells were grown in the presence of galactose before shifting to medium containing glucose for 48 h and spot testing at 30°C for 3 days. OD, attenuance. (**b**) Submitochondrial localization of Erv1 mutants compared with that of the WT. Mitochondria were isolated in the presence of glucose medium for 48 h. Isolated mitochondria were analysed by proteolytic susceptibility, SDS/PAGE and Western blotting against the indicated proteins. Cpn10, chaperonin 10; Cyt b2, cytochrome *b*_2_; Lysis, lysed mitochondria; Mito., intact mitochondria; M.P., mitoplasts; P.K., proteinase K; Tom40, translocase of the outer mitochondrial membrane 40 kDa.

Since we observed both mutants Erv1^SXXC^ and Erv1^CXXS^ not being able to support cell growth, we checked whether the mutation of the shuttle cysteine residues resulted in defective import of Erv1 into the mitochondria. To achieve this, we used a yeast strain with *ERV1* gene under the control of a *GAL10* promoter [31] transformed with plasmids encoding for WT or mutant Erv1 (Figure 3a). A strain with the empty vector (−) was used as a background control. Upon depletion of endogenous Erv1 in the presence of glucose, although the strain with plasmid-encoded WT Erv1 showed a normal growth phenotype, mutation of either one or both shuttle cysteine residues induced a cell growth defect as that observed for the background control strain at 30°C (Figure 3a). The low background level growth was probably due to a very low level of endogenous Erv1 which was sufficient to support growth. A similar result was observed at an elevated temperature of 37°C. At 37°C, there was a more pronounced growth defect for all the mutant and background control strains, whereas the growth of the WT strain was unaffected (results not shown). Thus these results are consistent with those obtained from using the ∆*erv1*-knockout strain (Figure 2), suggesting that both the N-terminal shuttle cysteine residues of Erv1 are required for cell growth.

**Figure 4:**
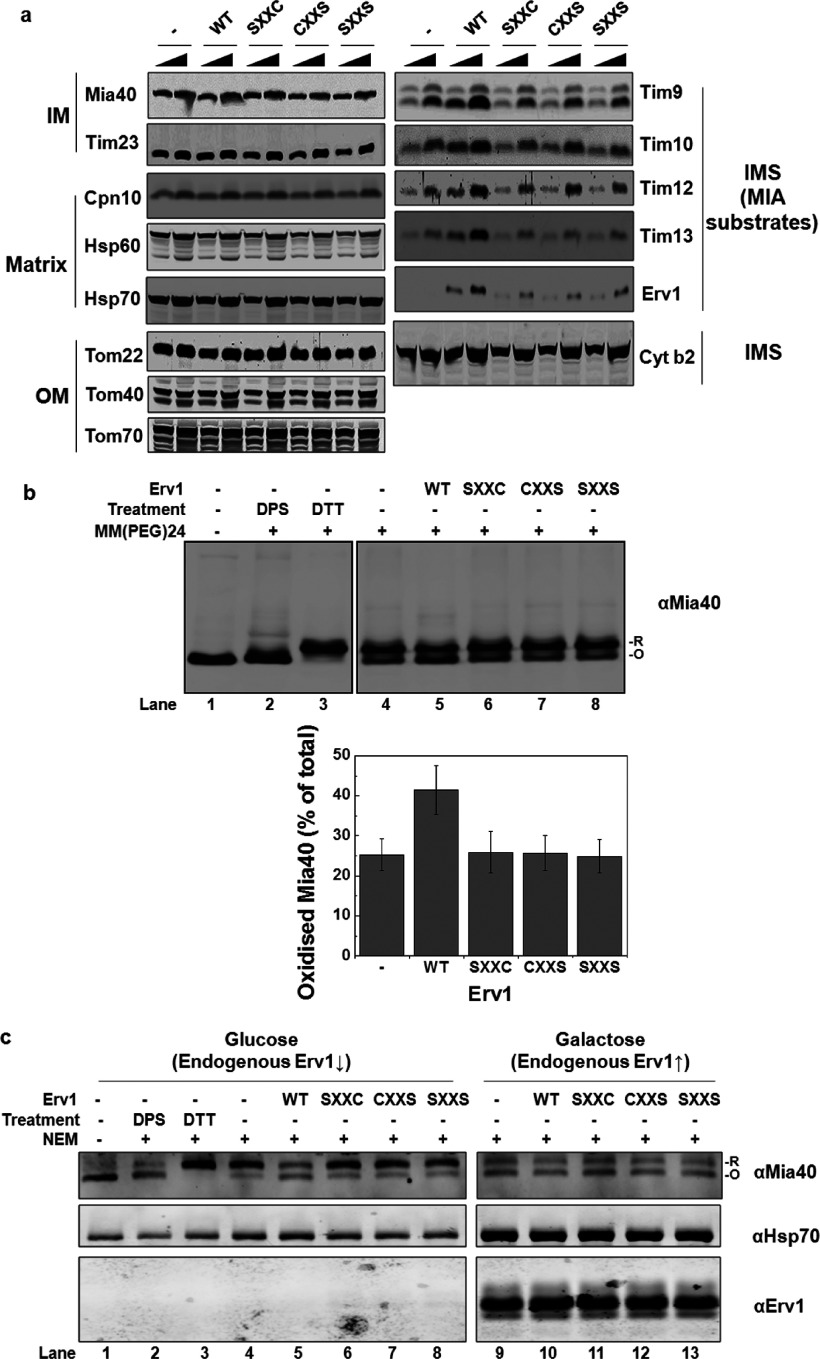
Mutation of the shuttle cysteine residues of Erv1 affected the function of MIA pathway (**a**) Steady-state level of proteins in mitochondria isolated from *P_GAL_*-*ERV1* strains (Figure 3a) with endogenous Erv1 depleted for 72 h. Different amounts of isolated mitochondria (50 and 100 μg) were loaded for comparison. (**b**) Redox state analysis of endogenous Mia40 in mitochondria. Mitochondria isolated as in (**a**) were resuspended in SDS/PAGE sample buffer in the presence or absence of 2 mM mmPEG24 and incubated at room temperature for 1 h before SDS/PAGE and Western blotting against Mia40. For controls (lanes 2–3), mitochondria were pre-treated with 5 mM DTT or 2 mM DPS (2,2′-dipyridyldisulfide) for 30 min to reduce or oxidize Mia40. The oxidized (O) and reduced (R) forms of Mia40 are indicated. Lower panel: the relative intensities of oxidized Mia40 in the WT and mutant strain mitochondria were quantified. Results are means±S.E.M., *n*=3. (**c**) Redox state analysis of the endogenous Mia40 *in vivo* using whole cell extracts. The WT and mutant cells were grown in glucose (lanes 1–8) or galactose (lanes 9–13) medium until the mid-exponential phase before incubation with 25 mM NEM on ice for 30 min, followed by total protein extraction and Western blotting with the anti-Mia40 antibody. The oxidized (O) and reduced (R) forms of Mia40 are indicated. The mitochondrial marker protein Hsp70 (heat-shock protein 70) was used as a loading control. Cpn10, chaperonin 10; Cyt b2, cytochrome *b*_2_; Tom, translocase of the outer mitochondrial membrane ↑, induce; ↓, repress.

The *P_GAL_-ERV1* strains carrying the Erv1 mutant plasmids allowed cell growth to a certain extent under the condition tested due to a very low undetectable level of endogenous Erv1 (see Figure 4a, lane -). This enabled us to isolate the mitochondria from the strains for further analyses. To check Erv1 localization in the mitochondria, we tested the proteolysis susceptibility of Erv1 and mitochondrial marker proteins in each of the mitochondrial subcompartments (Figure 3b). Tom40 (translocase of the outer mitochondrial membrane 40 kDa), cytochrome *b*_2_ and Cpn10 (chaperonin 10) were used as marker proteins in the mitochondrial OM (outer membrane), IMS and matrix respectively. Like cytochrome *b*_2_, the WT and mutant Erv1 proteins were detectable in intact mitochondria (Mito) but susceptible to protease diges-tion in mitoplasts (M.P.) when proteinase K was present (Figure 3b, lanes 1–4), indicating that the proteins were successfully imported into the mitochondrial IMS. Thus the shuttle cysteine mutations had no obvious effect on Erv1 localization, which is consistent with the observations that shuttle cysteine mutants had mild effects on the *in vitro* import of Erv1 [23,39].

In conclusion, yeast complementation assays and mitochondrial protein localization analyses show that both of the N-terminal shuttle cysteine residues are required for cell growth and thus the function of Erv1.

### Both of the N-terminal shuttle cysteine residues are required for the import of MIA substrates *in vivo*

After establishing the requirement of both Cys^30^ and Cys^33^ for Erv1 function, we then asked whether they are both required for the import of MIA substrates by checking the steady-state levels of proteins in the mitochondria isolated from the strains with depleted endogenous Erv1 (Figure 4a). In agreement with our results from the *in vivo* complementation assays, the protein level of MIA substrates (small Tim proteins and Erv1), which are essential for cell growth, was significantly decreased in mitochondria containing Erv1 with either one or both of the N-terminal shuttle cysteine residues mutated compared with that of the WT. As controls, the steady-state levels of other mitochondrial proteins were unaffected. These results indicated that the defect in the MIA pathway may be due to a functional defect of Erv1 mutants. However, we cannot conclude whether the effects were exclusively due to the cysteine mutations or to a combination of Erv1 abundance and compromised enzyme function.

To demonstrate the effect of the cysteine mutations on the MIA pathway more directly, we analysed the redox state of the Mia40 CPC motif in the mitochondria containing WT and mutant Erv1 (Figure 4b). In the presence of WT Erv1, more Mia40 (approximately 43%) was in an oxidized state (lane 5) compared with approximately 25% oxidized Mia40 in the mitochondria from the empty vector (lane 4) and mutant Erv1 strains (lanes 6–8), suggesting that the mutants had defects in oxidizing Mia40. Furthermore, we analysed the cysteine-redox state of Mia40 *in vivo* using whole cell extracts with NEM alkylation (Figure 4c) as described previously [12]. Consistently, upon depletion of endogenous Erv1 by growing the cells in glucose medium, approximately 50% Mia40 from the WT Erv1 strain was oxidized, but most of the proteins were reduced (~80%) in cells containing the empty vector or mutant Erv1 (Figure 4c, lanes 4–8). On the other hand, the same redox state distribution was observed for all strains when they were grown in galactose medium (lanes 9–13) when endogenous Erv1 was overexpressed. Western blot analysis of Erv1 showed that the same level of endogenous Erv1 was overexpressed in galactose medium. When the cells were grown in glucose medium, Erv1 was not detectable due to a low level of Erv1 expression [38].

Taken together, our findings suggest that both Cys^30^ and Cys^33^ are essential for Erv1 function in the MIA pathway, which is ultimately required for cell viability.

### Cys^33^ is involved in transferring electrons to the redox centre

**Figure 5:**
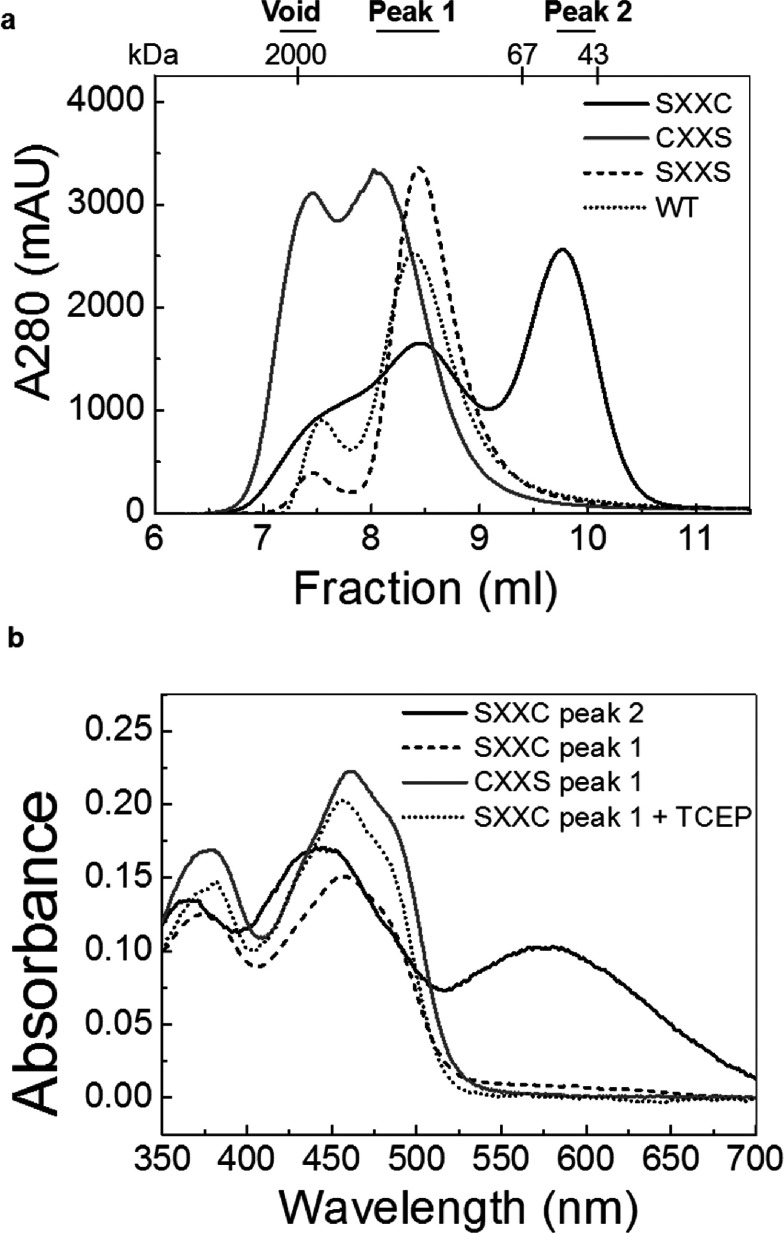
Characterization of purified Erv1 mutant proteins (**a**) Gel-filtration profiles of Erv1SXXC (black curve) and Erv1^CXXS^ (grey curve) compared with that of WT (dotted curve) the corresponding double cysteine mutant Erv1^SXXS^ (dashed curve) on Superdex 75 column equilibrated with buffer A [50 mM Tris/HCl buffer (pH 7.4) and 150 mM NaCl]. The molecular mass and the corresponding peak fractions are indicated. (**b**) UV–visible absorption spectra of the Erv1^SXXC^ and Erv1^CXXS^ mutant proteins. Peak fractions separated by size-exclusion chromatography in (**a**) were analysed: Erv1^SXXC^ peak 2 (black curve), Erv1^SXXC^ peak 1 (dashed curve), Erv1^CXXS^ peak 1 (grey curve) and Erv1^SXXC^ peak 2 treated with 2 mM of immobilized TCEP (dotted curve). All measurements were done in buffer A at 25°C.

To understand why both the shuttle cysteine residues are vital for Erv1 function, we investigated the function of individual cysteine residues *in vitro* using the recombinant single cysteine mutant proteins Erv1^SXXC^ (Erv1C30S) and Erv1^CXXS^ (Erv1C33S) expressed and purified from *E. coli* cells (see the Experimental section). Although purified Erv1^CXXS^ was yellowish as with the WT and the corresponding double cysteine mutant Erv1^SXXS^ [13], Erv1^SXXC^ was a black colour as reported previously [28]. We analysed the proteins further using gel-filtration chromatography (Figure 5a). Erv1^SXXC^ (C30S) eluted into two main peaks (black curve). Then, the molecular masses of the fractions were determined by light-scattering analysis to be approximately 99.8 kDa (peak 1) and 48.7 kDa (peak 2) respectively, suggesting that this mutant existed as both a tetramer and a dimer. Interestingly, the two peaks showed different colours, a WT-like yellow for peak 1 and black for peak 2. For Erv1^CXXS^ (C33S), two partially overlapping peaks were obtained eluted at the void volume (higher oligomers) and the position corresponding to the Erv1 tetramer (Figure 5a, grey curve).

UV–visible spectroscopy analysis (Figure 5b) revealed that both the yellowish species (peak 1) of Erv1^SXXC^ (C30S) and Erv1^CXXS^ (C33S) had a very similar spectra profile as that of the WT with a bound FAD absorbance at 460 nm. However, the black species (peak 2) of Erv1^SXXC^ (black curve) had a scattered increasing absorbance at the visible spectrum with a broadened peak at ~447 nm and an additional peak at ~580 nm, which is an indication of the presence of an S^−^-FAD CTC (charge-transfer complex) as reported previously [28]. Upon treatment with the reducing agent TCEP [tris-(2-carboxyethyl)phosphine], the spectra profile changed to the WT-like spectrum (Figure 5b, dotted curve), indicating that the intermediate CTC was resolved. We reasoned that the black-coloured Erv1^SXXC^ represents a charge-transfer intermediate containing a trapped disulfide bond between Cys^33^ and Cys^130^ of the active-site disulfide, leaving the unpaired Cys^133^ to form an S^−^-FAD CTC as seen in several flavoenzymes [16,28,40,41]. Upon TCEP treatment, the Cys^33^–Cys^130^ disulfide bond and the CTC were resolved, and the subsequent formation of the active-site Cys^130^–Cys^133^ disulfide bond led to the appearance of a WT-like absorption spectrum. No CTC was detected in Erv1^CXXS^, suggesting that it is unlikely for Cys^30^ to interact directly with the active-site disulfide bond. Thus Cys^33^ is functionally distinct from Cys^30^ in relaying electrons to the redox centre.

### Cys^30^ of Erv1 is more reactive in inter-molecular disulfide bond formation

**Figure 6:**
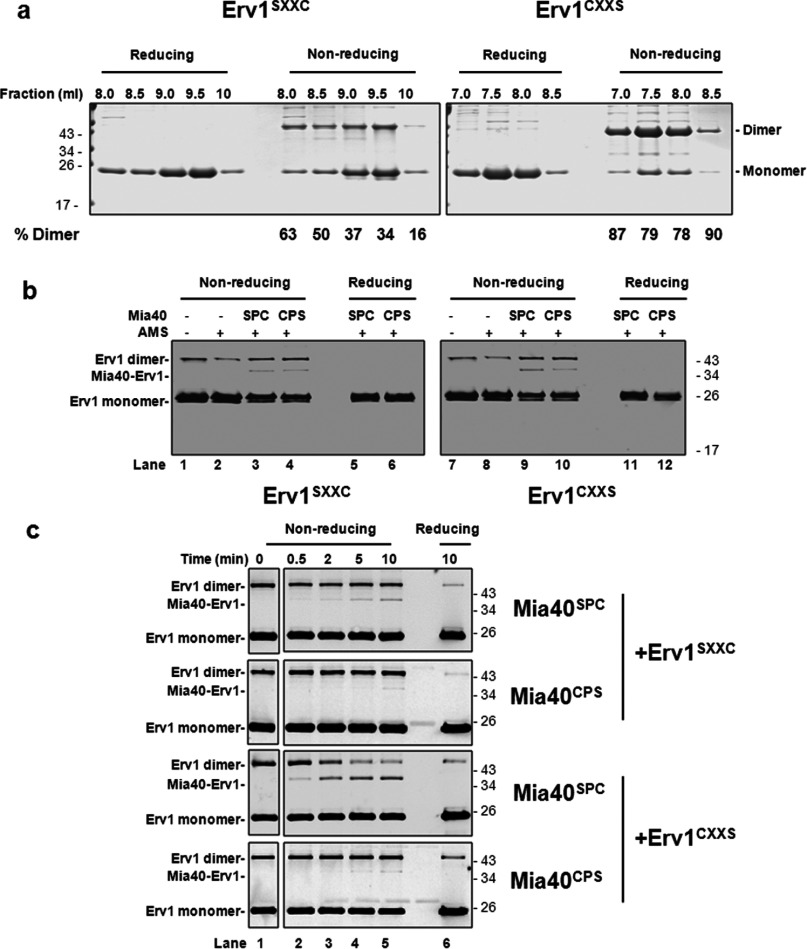
Cys^30^ is more reactive than Cys^33^ for intermolecular disulfide bond formation (**a**) Tris/Tricine SDS/PAGE (16% gel) of Erv1^SXXC^ and Erv1^CXXS^ mutant protein peak fractions in Figure 5(a) under reducing and non-reducing conditions. (**b**) Mixed disulfide bond formation between single cysteine mutants of Mia40 CPC (Mia40^SPC^ and Mia40^CPS^) and Erv1 shuttle cysteine residues (Erv1^SXXC^ and Erv1^CXXS^). The mutant proteins were pre-treated briefly with 2 mM TCEP and buffer-exchanged to buffer A before incubation at equimolar concentrations of 5 μM for 20 min at room temperature. The reactions were stopped by the addition of sample buffer with 1 mM DTT or 2.5 mM AMS. The proteins were detected by Western blotting with an antibody against Erv1. (**c**) Time courses of mixed disulfide bond formation between Mia40 and the Erv1 mutants. The proteins were treated as in (**b**) over a 10-min incubation. The molar ratio concentration of Mia40 to Erv1 used was 10:1 (50 μM Mia40 and 5 μM Erv1). The reactions were stopped at each time point by addition of sample buffer containing 20 mM IAM. As a reducing control, reactions at the end of each time course were analysed under reducing conditions by resuspending the reaction mixtures in sample buffer containing DTT.

The gel-filtration analysis (Figure 5a) showed that Erv1^CXXS^, in the presence of Cys^30^, was prone to form higher-molecular-mass oligomers, and the relative amount of the oligomers decreased significantly upon mutation of Cys^30^ in both the single and double mutants. This result suggested that Cys^30^ may be more solvent exposed and plays a more prominent role than Cys^33^ in forming the intermolecular disulfide bond. To test this, we analysed the gel-filtration fractions of the purified proteins using reducing and non-reducing SDS/PAGE (Figure 6a). As predicted, the results showed that all of the fractions of Erv1^CXXS^ were dominated by an intermolecular disulfide-bonded dimer and more prone to form such a dimer than Erv1^SXXC^. Such a dimer band was observed for the WT, redox active-site and struc-tural disulfide double cysteine mutants (C130/C133S and C157/C176S), but not detectable or negligible for the double shuttle cysteine mutant (C30/C33S) (Supplementary Figure S1 at http://www.biochemj.org/bj/460/bj4600199add.htm), suggesting that the dimer band in Figure 6(a) contained mainly Erv1 dimers linked via Cys^30^–Cys^30^′ or Cys^33^–Cys^33^′ intermolecular disulfide bond respectively. Thus the higher reactivity of Cys^30^ compared with that of Cys^33^ for intermolecular disulfide bond formation between Erv1 molecules gave an early indication that Cys^30^ may play an important role in receiving electrons from protein thiols of substrate molecules.

Next, to understand whether Cys^30^ of Erv1 is more reactive than Cys^33^ in interacting with the Mia40 CPC motif, Erv1^SXXC^ and Erv1^CXXS^ were incubated with single cysteine mutants of the core domain Mia40 (~15 kDa; see the Experimental section) CPC motif disulfide (hereafter named Mia40^SPC^ and Mia40^CPS^) respectively, in all four possible combinations and then analysed using non-reducing SDS/PAGE. Although an adduct of the ~37 kDa band was observed in all four combinations (Figure 6b, lanes 3, 4, 9 and 10), Erv1^CXXS^ plus Mia40^SPC^ showed the most intensive band (lane 9). This adduct was dissociated under reducing conditions (Figure 6b, lanes 5, 6, 11 and 12), showing that the 37 kDa adduct was an Mia40–Erv1 complex containing an intermolecular disulfide bond between the mutant proteins (15 kDa Mia40 and 22 kDa Erv1). The highest amount of the complex was detected in the mixture of Erv1^CXXS^ and Mia40^SPC^ (Figure 6b, lane 9), implying that the mixed disulfide formed between the first cysteine residue (Cys^30^) of Erv1 and the second cysteine of the Mia40 CPC motif is the most favourable. This result was confirmed and shown more clearly by time course analyses (Figure 6c). The complex formed between of Erv1^CXXS^ (Cys^30^) and Mia40^SPC^ was most prominent and effective compared with the other three combinations (Figure 6c, lanes 2–5). Taken together, our results suggest that Cys^30^ is more reactive than Cys^33^ in forming an intermolecular disulfide bond with Mia40, and more reactive towards the second cysteine residue of Mia40 CPC.

In human ALR, however, this was not the case, as both of its shuttle cysteine residues had an equal level of interaction with the second cysteine of the hMia40 CPC motif based on cysteine mutation and non-reducing gel analyses [21]. Furthermore, detailed structural characterization of the ALR–hMia40 covalent adducts suggested that the second shuttle cysteine residue of ALR (analogous to Erv1 Cys^33^ rather than Cys^30^) may interact preferentially with hMia40 attributed to the presence of hydrophobic residues downstream of the second shuttle cysteine residue (bold) of ALR (CRA**C**VDFKTWM) [21]. Similar, but different, hydrophobic residues are present downstream of Erv1 Cys^33^ (bold) (CRS**C**NTLLDFQ). A previous computational simulation suggested that Cys^33^ of Erv1 may form an intermolecular disulfide bond with Mia40 driven by hydrophobic interactions [17]. It is worth to mention that the domains containing the shuttle cysteine residues of Erv1 and ALR are not conserved and both are largely unstructured. A recent structural study has suggested that the shuttle cysteine residues of Erv1 and ALR have different structural and solvent exposal properties [42]. Although both shuttle cysteine residues of ALR are located in a flexible segment, Cys^33^ of Erv1 seems to be part of a α-helical structure with Cys^30^ unstructured and thus more solvent exposed [42]. This result is consistent with our observation that Cys^30^ is more reactive than Cys^33^ in forming intermolecular disulfide bond between Erv1–Erv1′ and with Mia40.

**Figure 7:**
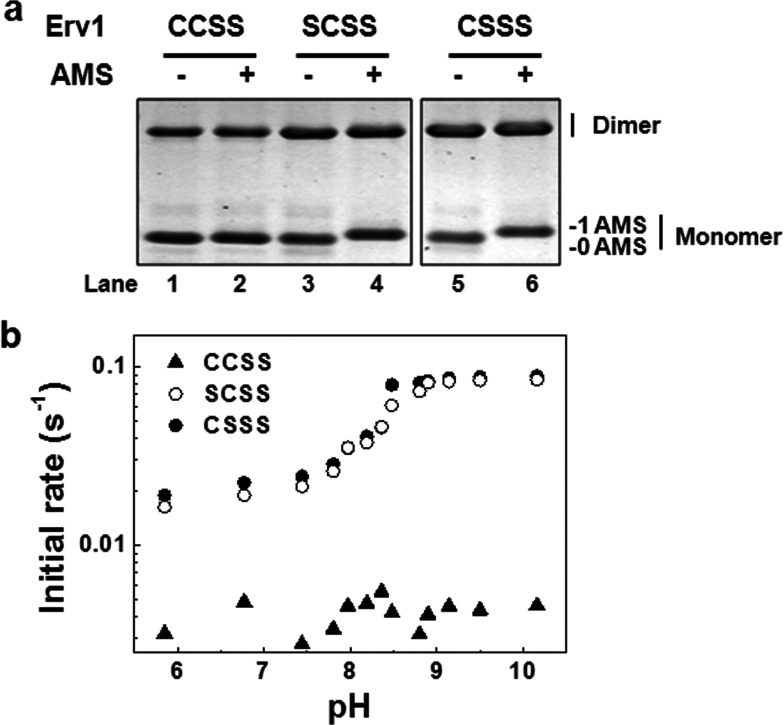
p*K*_a_ determinations for Cys^30^ and Cys^33^ of Erv1 (**a**) Redox state analysis of protein thiols. Purified proteins were treated with or without 2.5 mM AMS in the dark at room temperature for 30 min before analysis by SDS/PAGE. A molecular mass increment of ~0.5 kDa as a result of AMS alkylation to free thiol is indicated. (**b**) The pH profile of the reaction rate for Erv1 mutant proteins with mBBr. Erv1 (2 μM) was incubated with mBBr (2 μM) at various pH values at 25°C and the fluorescent emission at 482 nm (λ_ex_=396 nm) were followed for 30 min. The points for at least the first 10 min were used to calculate the initial slope. The best fit p*K*_a_ values are 8.39±0.12 and 8.41±0.06 for Cys^30^ and Cys^33^ respectively. Erv1^CCSS^ was used as a control for Erv1 in the absence of free thiols.

Next, we asked whether the higher reactivity of Cys^30^ of Erv1 is due to the fact that it has a lower p*K*_a_ value than Cys^33^. In order to determine the p*K_a_* of both shuttle cysteine residues of Erv1, we generated two triple cysteine mutants from the single cysteine mutants Erv1^CXXS^ and Erv1^SXXC^, in which both active-site cysteine were mutated to serine (named as Erv1^CSSS^ and Erv1^SCSS^) to avoid potential thiol–disulfide exchange reactions. AMS thiol-modification confirmed that both Cys^30^ and Cys^33^ were reduced and accessible to a thiol alkylating agent (Figure 7a). Time courses of the interaction of the triple mutant with mBBr combined with fluorescence detection were measured at various pHs and the initial rates were determined. As a control, the redox active-site disulfide mutant Erv1^CCSS^ with the shuttle cysteine residues oxidized (Figure 7a, lane 2) was used. The results showed that both Cys^30^ and Cys^33^ have the same apparent p*K*_a_ values of 8.4±0.1 (Figure 7b). Thus the higher reactivity of Cys^30^ than that of Cys^33^ for disulfide bond formation was not due to a low p*K_a_*.

### Both Cys^30^ and Cys^33^ of Erv1 are required for enzyme turnover

**Figure 8:**
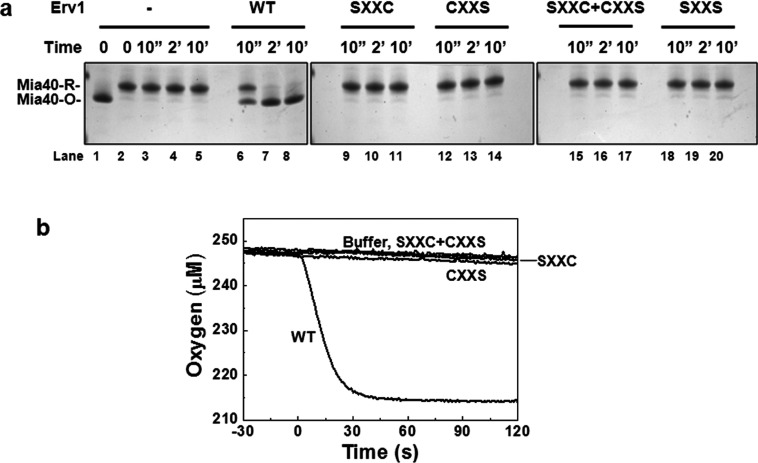
Both of the shuttle cysteine residues are required for oxidase function of Erv1 (**a**) Time-course analysis of the redox state change of 50 μM Mia40 with the CPC motif disulfide reduced in the absence or presence of a total of 2 μM of WT or cysteine mutant Erv1 either individually or in a mixture (SXXC+CXXS) at 25°C. Reactions at designated time points were quenched by 2.5 mM AMS. (**b**) Oxygen consumption profiles of oxidation of the CPC motif of reduced Mia40 (50 μM) in the presence of 2 μM of WT or single cysteine mutant Erv1 as described above.

To understand further whether and why both Cys^30^ and Cys^33^ are required for the oxidase activity of Erv1, we analysed the oxidase activity of Erv1 mutants using gel-based AMS thiol modification and oxygen consumption assays using purified reduced Mia40 as a substrate [13]. First, as shown in Figure 8(a), Mia40 was fully oxidized within 2 min of incubation with the WT Erv1 (lanes 6–8). However, Mia40 stayed in a reduced form throughout the time course when incubated with Erv1^SXXC^ and Erv1^CXXS^ respectively (Figure 8a, lanes 9–14), or with both mutants together (lanes 15–17). Next, Erv1 activity was measured by oxygen consumption (Figure 8b). Consistently, only the WT Erv1 was able to catalyse the electron transfer from Mia40 to molecular oxygen, but not the mutants alone or in a combination. These results showed that both Cys^30^ and Cys^33^ are required, and must be in the same CXXC motif (not CXXS+SXXC) for Erv1 enzyme activity turnover.

Theoretical and computational studies have suggested that the thiol–disulfide exchange reaction proceeds through a S_N_2 transition state, and steric restriction plays a significant role in such a reaction [43,44]. The thiol–disulfide exchange reaction proceeds optimally when the attacking thiolate and the disulfide bond are in a co-linear position [43,44]. Consistently, our results showed that Cys^30^ of Erv1 in the same CXXC motif with Cys^33^ is essential for Erv1 enzyme activity. We reasoned that Cys^30^ is required for the intermediate disulfide Cys^33^–Cys^130^ to be resolved rapidly allowing the active enzyme be regenerated effectively. Taken together, these results showed that both shuttle cysteine residues are required for Erv1 oxidase activity and provided an explanation why both the single shuttle cysteine mutations caused cell growth defect (Figures 2 and 3a).

### Conclusion

**Figure 9:**
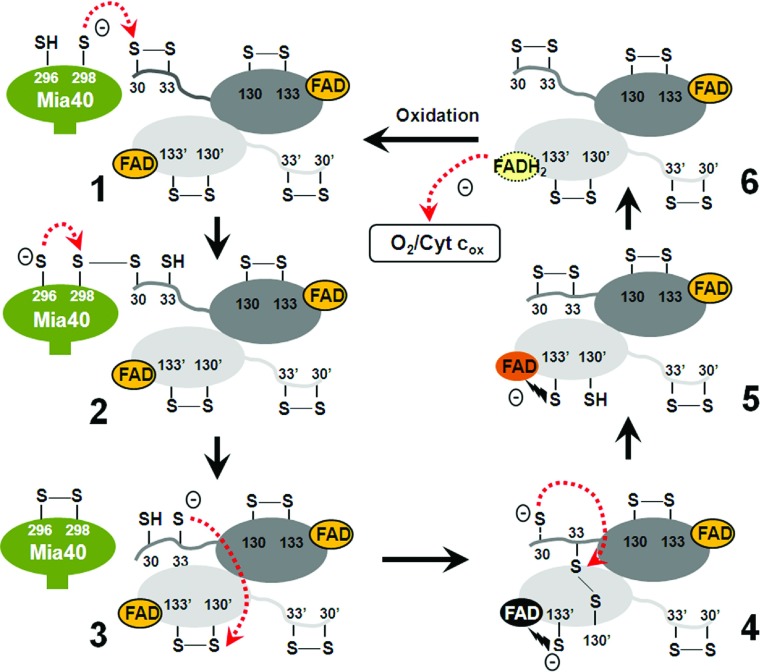
Model of thiol–disulfide exchange reactions in Erv1 catalysis This model is focused on the reductive half-reaction. Thiolate anion (S^−^) of the second cysteine (Cys^298^) of the Mia40 CPC motif nucleophilically attacks Cys^30^ of Erv1 (stage 1), leading to the formation of Mia40–Erv1 mixed disulfide bond, which is then attacked by S^−^ of the first cysteine (Cys^296^) of Mia40 for oxidation of the CPC motif and reduction of the Erv1 Cys^30^–Cys^33^ disulfide bond (stage 2). Thiolate of Cys^33^ nucleophilically attacks Cys^130^′ of the other subunit of Erv1 (stage 3), leading to the formation of an intermediate Cys^33^–Cys^130^′ disulfide bond between two Erv1 subunits and a thiolate to FAD CTC (stage 4). Then, the Cys^33^–Cys^133^′ disulfide is reduced via nucleophilic attack on Cys^33^ by Cys^30^ (step 4), which leads to reoxidation of the Cys^30^–Cys^33^ disulfide bond (stage 5). Subsequently, a pair of electrons from the reduced redox active-site Cys^130^′/Cys^133^′ is transferred to FAD coupled with the reformation of the Cys^130^′–Cys^133^′ disulfide bond (stage 6). Finally, the oxidative half-reaction for Erv1 turnover is completed through transfer of a pair of electrons from reduced FAD (FADH_2_) to molecular oxygen or cytochrome *c*. The colour of FAD in stages 4 and 5 correspond to the colour of Erv1C30S and Erv1C130S respectively (the present study and [28]).

In the present study, we have shown that both shuttle cysteine residues of Erv1 are required for Erv1 function and that they each play distinct roles in the Mia40–Erv1 enzymatic mechanism. Together with the results of previous studies [13,17,28], we propose a model for Erv1 thiol–disulfide exchange reactions with a focus on the reductive half-reaction (Figure 9). Cys^30^ of Erv1 is much more reactive than Cys^33^ in forming an intermolecular disulfide bond with the second cysteine residue of the Mia40 CPC motif, thus Cys^30^ is the preferred cysteine to interact directly with Mia40 in the Mia40–Erv1 reaction. In the Erv1–Erv1′ electron transfer reaction, Cys^33^ is essential for forming the intermediate disulfide Cys^33^–Cys^130^′ (where Cys^130^′ is the interchange cysteine of another subunit), whereas Cys^30^ is required for resolving the intermediate disulfide for efficient turnover of the enzyme. We propose that Cys^30^ of Erv1 plays two important roles: interacting with Mia40 and resolving the Erv1 intermediate disulfide Cys^33^–Cys^130^′ effectively. Taken together, both shuttle cysteine residues of Erv1 are required for its function, and they play complementary, but distinct, roles to ensure rapid turnover of Erv1 enzyme activity both *in vivo* and *in vitro*. In addition, the higher versatility of shuttle cysteine motifs and domains, and different levels of functional specificity between the shuttle cysteine residues in ERV/ALR enzymes highlight an evolutionary divergence of ERV/ALR homologues.

## Online data

Supplementary data
