# Supplementary material for: Mitochondrial thiol oxidase Erv1: both shuttle cysteine residues are required for its function with distinct roles
Source: Biochem J. 2014 May 13;460(Pt 2):199–210. doi: 10.1042/BJ20131540 (PMC4019985; doi:10.1042/BJ20131540)
Supplement: Supplementary data [file bj4600199add.pdf]

Biochem. J. (2014) **460**, 199–210 (Printed in Great Britain) doi:10.1042/BJ20131540

SUPPLEMENTARY ONLINE DATA

Mitochondrial thiol oxidase Erv1: both shuttle cysteine residues are required for its function with distinct roles

Swee Kim ANG*, Mengqi ZHANG*, Tiziana LODI† and Hui LU*[1]

*Manchester Institute of Biotechnology, Faculty of Life Sciences, University of Manchester, 131 Princess Street, Manchester M1 7DN, U.K.
†Department of Life Sciences, University of Parma, I-43100 Parma, Italy

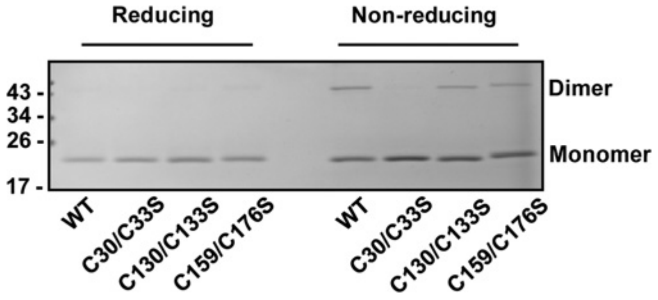

Figure S1 SDS/PAGE of Erv1 WT and double cysteine mutant proteins under reducing and non-reducing conditions

C30/C33S, shuttle disulfide mutant (SXXS); C130/C133S: active-site disulfide mutant; C159/C176S: structural disulfide mutant. Molecular mass is given on the left-hand side in kDa.

Received 25 November 2013/28 February 2014; accepted 13 March 2014
Published as BJ Immediate Publication 13 March 2014, doi:10.1042/BJ20131540

[1] To whom correspondence should be addressed email (hui.lu@manchester.ac.uk).

© 2014 The Author(s)
The author(s) has paid for this article to be freely available under the terms of the Creative Commons Attribution Licence (CC-BY) (http://creativecommons.org/licenses/by/3.0/) which permits unrestricted use, distribution and reproduction in any medium, provided the original work is properly cited.

Biochemical Journal www.biochemj.org
